# Association between serum uric acid levels and white matter hyperintensities in patients with mild ischemic stroke

**DOI:** 10.3389/fneur.2025.1590408

**Published:** 2025-07-21

**Authors:** Chaoxin Chai, Yuqi Cui, Meng Li, Jianing Xia, Yiming Wang, Fuyun Ren, Liping Chen

**Affiliations:** ^1^Department of Neurology, The Second Hospital of Hebei Medical University, Shijiazhuang, Hebei, China; ^2^Key Laboratory of Clinical Neurology (Hebei Medical University), Ministry of Education, Shijiazhuang, Hebei, China; ^3^Neurological Laboratory of Hebei Province, Shijiazhuang, Hebei, China

**Keywords:** serum uric acid, mild ischemic stroke, white matter hypersignaling, periventricular hyperintensity (PVH), deep white matter hyperintensities

## Abstract

**Objective:**

This study aims to investigate the association between uric acid (SUA) level and white matter hyperintensities (WMH) in patients with mild ischemic stroke.

**Methods and results:**

This study retrospectively analyzed 290 patients with mild ischemic Stroke who were hospitalized at the Second Hospital of Hebei Medical University from March 2021 to January 2022. General clinical information and laboratory test results were collected. WMH was interpreted on MRI, and periventricular hyperintensity (PVH) and deep white matter hyperintensities (DWMH) were scored, respectively. According to the score of Fazekas, WMH was divided into two groups. Factors influencing WMH severity were analyzed, and the relationship between SUA and WMH was further explored. The results showed that the SUA of patients with moderate to severe DWMH was higher than that of patients with no or mild DWMH (345.10 ± 97.52 μmol/L vs. 317.99 ± 91.21 μmol/L, *p* = 0.028). There was no significant difference in SUA between the moderate to severe PVH group and the no or mild PVH group (336.49 ± 99.29 μmol/L vs. 319.16 ± 89.92 μmol/L, *p* = 0.131). Spearman correlation analysis showed that SUA was positively correlated with the severity of DWMH (*r* = 0.123, *p* = 0.037), while SUA was not significantly correlated with PVH severity (*r* = 0.070, *p* = 0.234). After adjusting for confounders by multifactor logistic regression, SUA was independently correlated with DWMH severity (OR: 1.005, 95% CI: 1.002–1.009, *p* = 0.004). There was no significant correlation between SUA and PVH (OR: 1.003, 95% CI: 1.000–1.006*, p* = 0.051).

**Conclusion:**

SUA is an independent risk factor for DWMH in patients, and there is no significant correlation between SUA level and PVH.

## 1 Introduction

With the increasing prevalence of aging populations, aging-related diseases pose significant challenges to society. Advances in neuroimaging techniques have led to a rise in the detection of white matter lesions (WMLs), also referred to as white matter hyperintensities (WMH). WMH appears hyperintense on T2-weighted imaging (T2WI) and fluid-attenuated inversion recovery (FLAIR) sequences on MRI, while showing iso- or slightly hypointense signals in T1-weighted imaging (T1WI), though still higher in intensity than cerebrospinal fluid (CSF) (1). WMH is typically classified into periventricular hyperintensity (PVH) and deep white matter hyperintensities (DWMH), depending on the lesion’s anatomical location. Numerous studies have established a close association between WMH and conditions such as cognitive impairment (2), depression (3), and stroke (4). Despite these findings, the pathogenesis and risk factors of WMH remain unclear, with the underlying pathophysiological mechanisms varying by lesion location. Research (5, 6) has suggested that vascular factors, including blood–brain barrier disruption, alterations in capillary permeability, and reductions in regional cerebral blood flow (7–10), are primary contributors to WMH development. Additionally, venous collagen diseases may also play a role in WMH formation. Small-vessel atherosclerosis has been identified as a key contributor to DWMH, while PVH is more often associated with chronic cerebral hemodynamic insufficiency (11–14). Currently, no effective treatments exist to reverse WMH progression, making it clinically significant to further investigate the pathogenesis and risk factors for WMH in order to inform prevention and therapeutic strategies.

Uric acid (SUA) is the final metabolite of purine nucleotides and has the dual effects of antioxidation and promoting oxidation. Some studies have shown that UA can exert neuroprotective effects as a free radical scavenger. In the experimental model of stroke, UA can reduce ischemic injury and improve prognosis. Recent studies have shown that hypouricemia may lead to the development of stroke and is a risk factor for cerebrovascular diseases and all-cause mortality (15, 16). However, a meta-analysis showed that hyperuricemia was positively correlated with the risk of stroke (17). Elevated SUA may lead to cerebrovascular diseases through multiple mechanisms, including impaired NO production, endothelial dysfunction, increased vascular stiffness, elevated oxidative stress, endothelial cell apoptosis, disruption of the blood–brain barrier, and vascular fibrosis (18). These mechanisms can also lead to cerebral small vessel diseases, including the occurrence and development of WMH. Except for a few studies that suggest no correlation between SUA and WMH (19), the results of the majority of other studies indicate that high SUA levels are correlated with WMH. A (20) study found that SUA levels were only associated with the severity of DWMH in men, and the results of multivariate logistic regression showed that high SUA levels were an independent risk factor for moderate to severe DWMH. However, SUA levels had no significant correlation with DWMH in women, nor were they significantly correlated with PVH in either men or women. However, the results of other (21) study showed that after adjusting for confounding factors, high SUA levels were independently associated with severe PVH, especially in female patients, while SUA levels were not related to DWMH. Another research result (22) suggested that high SUA levels are independently associated with the severity of WMH, whether in DWMH or PVH. To sum up, although SUA has been implicated in both protective and harmful roles in cerebrovascular pathology, its site-specific relationship with WMH subtypes—particularly in mild ischemic stroke patients—has not been clearly elucidated. We therefore aimed to investigate the association between SUA and WMH, stratified by PVH and DWMH, in a cohort of mild ischemic stroke patients.

## 2 Methods

### 2.1 Object of study

The study is a retrospective observational cohort study. We retrospectively collected patients with mild ischemic stroke who were hospitalized at the Second Hospital of Hebei Medical University from March 2021 to January 2022.

Enrollment criteria: (1) Age range 18–80 years; (2) ischemic cerebrovascular events occurred within 2 weeks, NIHSS score ≤3 points; (3) brain MRI scan was performed; (4) SUA test was performed within 7 days after the occurrence of ischemic cerebrovascular events.

Exclusion criteria: (1) patients with Parkinson’s disease, dementia, severe traumatic, toxic or infectious brain injury, or brain tumor; (2) patients with severe heart disease and recent myocardial infarction or angina pectoris, severe infection, severe kidney or liver disease, thrombotic disease, and tumors; (3) taking urico-lowering or estrogenic drugs within 1 week of SUA collection; (4) poor image quality; (5) incomplete clinical data.

### 2.2 Collection of clinical data

Clinical data were gathered on patients enrolled in the study, encompassing key demographic and clinical parameters including age, gender, and medical history of hypertension, diabetes, smoking, and alcohol consumption. Neurological status was quantitatively assessed using the National Institutes of Health Stroke Scale (NIHSS). Comprehensive laboratory evaluations were conducted to measure lipid profiles—including total cholesterol (TC), high-density lipoprotein (HDL), low-density lipoprotein (LDL), and triglycerides (TG)—as well as fasting plasma glucose (FPG), glycosylated hemoglobin (HbA1c), homocysteine (Hcy), high-sensitivity C-reactive protein (hs-CRP), and serum uric acid (SUA).

### 2.3 MRIs protocol

Brain MR imaging was performed on a 3.0 Tesla MR scanner (Discovery 750, GE Healthcare, Milwaukee, United States) with an eight-channel head coil. The MR imaging protocol includes: T1-weighted imaging (T1WI), T2-weighted imaging (T2WI), fluid-attenuated inversion recovery (FLAIR), and diffusion-weighted imaging (DWI). The imaging parameters for the brain routine MR imaging were: T1WI: FSE, TR/TE 2,000/10 ms, FOV 24 × 24 cm^2^, spatial resolution 0.9 × 0.9 mm^2^, slice thickness 5 mm, total scan time 1 min 29 s; T2WI: FSE, TR/TE 5,700/97 ms, FOV 24 × 24 cm^2^, spatial resolution 0.9 × 0.9 mm^2^, slice thickness 5 mm, total scan time 1 min 12 s; FLAIR: inversion recovery (IR), TR/TE 9,000/150 ms, FOV 24 × 24 cm^2^, spatial resolution 0.9 × 0.9 mm^2^, slice thickness 5 mm, total scan time 1 min 49 s; DWI: echo-planar imaging, TR/TE 3,000/65 ms, FOV 24 × 24 cm^2^, spatial resolution 1.6 × 1.6 mm^2^, slice thickness 5 mm, total scan time 42 s.

### 2.4 Assessment and grouping of WMH severity

**Figure 1:**
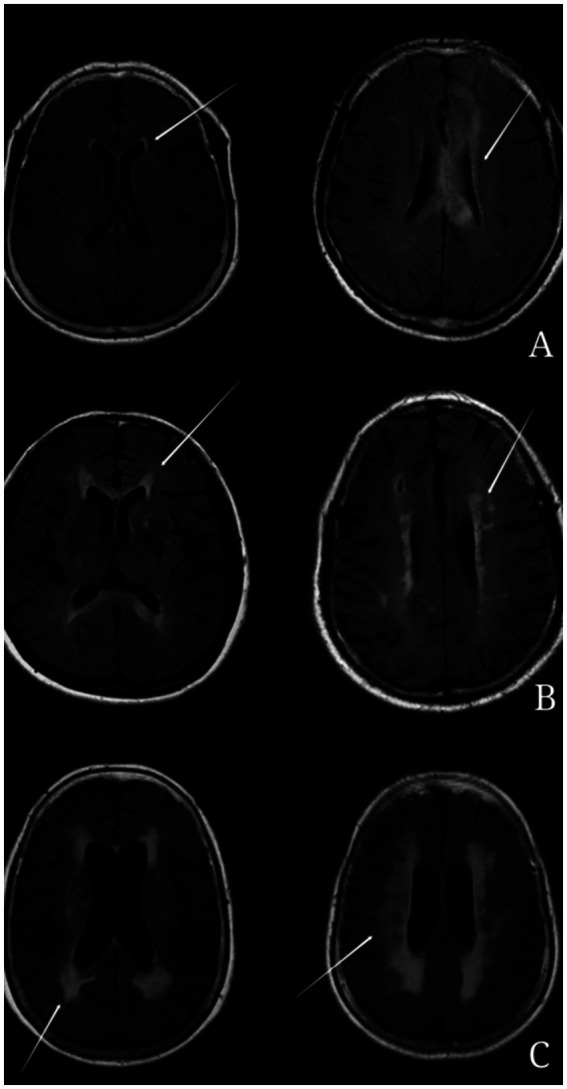
Example of periventricular hyperintensity (PVH). **(A)** 1 point: thin-layer lesions of dotted line or cap shape near the anterior and posterior feet of the lateral ventricle. **(B)** 2 points: thick cap like or halo like lesions. **(C)** 3 points: the degree of white matter lesion is severe, even extending to subcortical white matter.

**Figure 2:**
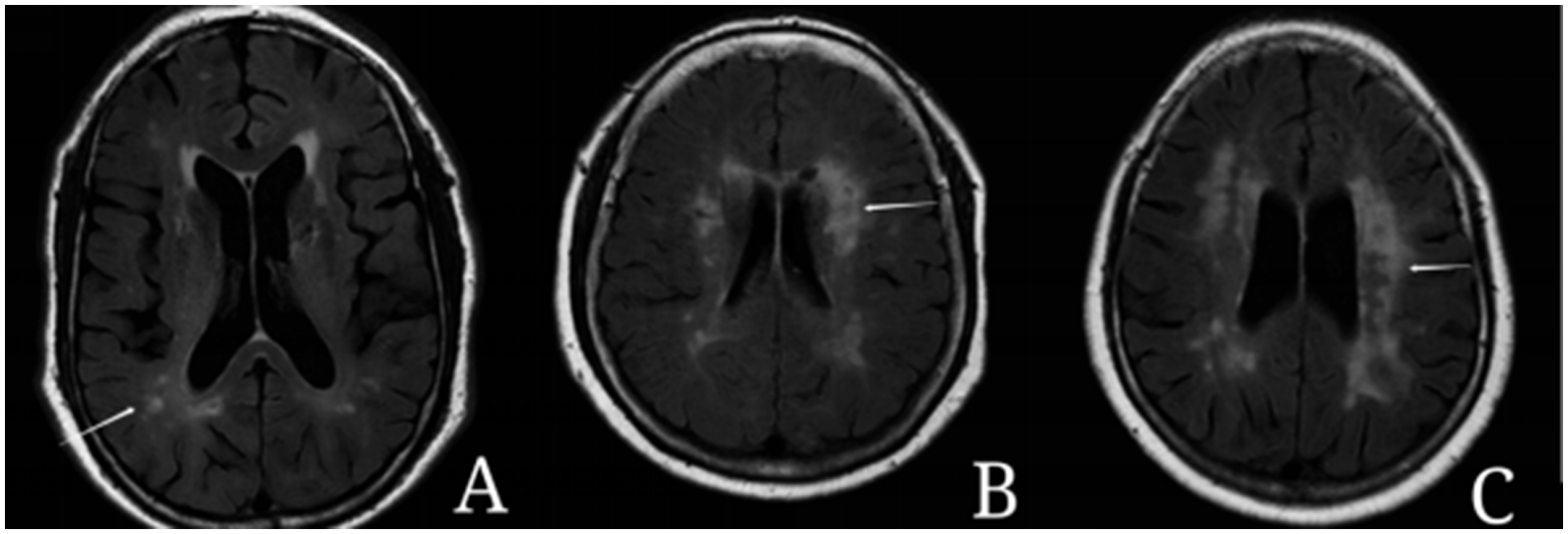
Example of deep white matter hyperintensities (DWMH). **(A)** 1 point: the lesions show punctate changes. **(B)** 2 points: some punctate lesions begin to fuse. **(C)** 3 points: the lesions fuse in a large area.

The assessment and classification of white matter hyperintensities (WMH) were conducted using the Fazekas scale (23). This scale categorizes WMH into:

1. Periventricular hyperintensities (PVH) (Figure 1):
  - Score 0: Absence of hyperintense signals.
  - Score 1: Presence of linear or thinly capped lesions.
  - Score 2: Thick capping or halo-like lesions.
  - Score 3: Irregular and severe lesions extending into the subcortical white matter.
2. Deep white matter hyperintensities (DWMH) (Figure 2):
  - Score 0: No high signal abnormalities.
  - Score 1: Isolated focal changes.
  - Score 2: Beginning fusion of focal lesions.
  - Score 3: Extensive area of lesion fusion.

WMH severity was thus stratified into two categories based on their score: mild (0 or 1) and moderate to severe (2 or 3). Imaging assessments were independently performed by two trained neuroimaging physicians blinded to the participants’ clinical information. Discrepancies between evaluators were resolved through additional review by a senior neurologist, who also remained blinded to the initial imaging results.

### 2.5 Statistical methods

Continuous variables were characterized using either mean ± standard deviation or median and interquartile range, while categorical variables were described by frequency (percentage). The inter-rater reliability of WMH assessment results among evaluators was assessed using Cohen’s kappa coefficient. The analysis of continuous variables was conducted employing either the independent sample *t*-test or the Mann–Whitney *U* test, depending on the distribution of the data. Categorical variables were analyzed using the chi-square test. The association between serum uric acid (SUA) levels and the severity of white matter hyperintensities (WMH) was investigated using Spearman correlation analysis. Furthermore, both univariate and multivariate logistic regression analyses were utilized to explore the relationship between SUA levels and WMH severity, with the computation of odds ratios (OR) and 95% confidence intervals (CI). In multivariate logistic regression, confounders with *p*-values <0.05 in the single factor analysis were adjusted. All statistical analyses were performed using SPSS version 24.0 (SPSS, Inc., San Diego, CA). A *p*-value of less than 0.05 was deemed indicative of statistical significance.

## 3 Results

### 3.1 Baseline characteristics of study participants

A total of 290 patients were included in the study, of which 201 were male (69.3%) and 89 were female (30.7%). The patients were divided into two groups according to the severity of WMH in different parts: no PVH or mild PVH group 186 cases (64.1%), moderate to severe PVH group 104 cases (35.9%); There were 211 cases (72.8%) in the DWMH no or mild group and 79 cases (27.2%) in the moderate to severe group. Clinical information and laboratory test results for all patients and subgroups are summarized in Tables 1, 2. Patients in the moderate to severe group were older than those in the mild group for both DWMH and PVH (DWMH: 61.34 ± 9.24 vs. 51.26 ± 12.49, *p* < 0.001; PVH: 60.63 ± 10.37 vs. 50.30 ± 12.10, *p* < 0.001) had a higher incidence of hypertension (DWMH: 119 cases, 82.3% vs. 77 cases, 62.1%, *p* < 0.001; PVH: 86 cases, 82.7% vs. 110 cases, 59.1%, *p* < 0.001), homocysteine [DWMH: 15.2 (11.5–22.4) μmol/L vs. 12.1 (9.2–16.4) μmol/L, *p* < 0.*001*; PVH: 15.7 (11.5–23.15) μmol/L vs. 11.55 (9.1–16.1) μmol/L, *p* < 0.001], hypersensitive C-reactive protein [DWMH: 2.3 (1.2–4.6) mg/L vs. 1.8 (1–3.8) mg/L, *p* = 0.045; PVH: (1.2–4.38 mg/L) 2.15 vs. 1.8 (1–3.8 mg/L, *p* = 0.045) higher levels. For DWMH, the SUA level was higher in the moderate to severe group than in the mild group (345.10 ± 97.52 μmol/L vs. 317.99 ± 91.21 μmol/L, *p* = 0.028). There was no significant difference in SUA level between the moderate to severe PVH group and the no or mild PVH group (336.49 ± 99.29 μmol/L vs.319.16 ± 89.92 μmol/L, *p* = 0.131).

**Table 1:** Comparison between DWMH no or mild group and moderate and severe DWMH group.

Variables	Total number of cases (*n* = 290)	No or light (*n* = 211)	Moderate-severe (*n* = 79)	Check value	*p*-value
Age (years)	54.01 ± 12.52	51.26 ± 12.49	61.34 ± 9.24	−7.437	<0.001
Male [ex (%)]	201 (69.3)	143 (67.8)	58 (73.4)	0.861	0.353
History of hypertension [cases (%)]	196 (0.676)	131 (62.1)	65 (82.3)	10.699	0.001
History of diabetes [cases (%)]	77 (26.6)	57 (27.0)	20 (25.3)	0.085	0.771
Smoking history [ex. (%)]	136 (46.9)	97 (46.0)	39 (49.4)	0.266	0.606
Drinking history [ex. (%)]	91 (31.4)	68 (32.2)	23 (29.1)	0.259	0.611
NIHSS rating	1.67 ± 1.03	1.62 ± 1.03	1.81 ± 1.00	−1.436	0.152
Infarct site [anterior circulation/case (%)]	193 (66.6)	140 (66.4)	53 (67.1)	0.014	0.906
TG (mmol/L)	1.61 ± 0.78	1.65 ± 0.80	1.51 ± 0.73	1.332	0.184
HDL (mmol/L)	1.05 ± 0.28	1.04 ± 0.27	1.07 ± 0.29	−0.510	0.610
LDL (mmol/L)	2.65 ± 0.91	2.70 ± 0.93	2.49 ± 0.82	1.825	0.069
FPG (mmol/L)	5.31 (4.87 to 6.77)	5.32 (4.9–6.92)	5.22 (4.72–6.32)	−0.324	0.277
HbA1c (%)	6 (5.4–7.03)	6 (5.5–7.2)	6 (5.3 to 6.9)	−0.405	0.428
hs-CRP (mg/L)	1.91 (1.1 to 3.9)	1.8 (1 to 3.8)	2.3 (1.2 to 4.6)	−1.561	0.045
Hcy (μmol/L)	13 (9.7 to 17.63)	12.1 (9.2 to 16.4)	15.2 (11.5–22.4)	−2.971	<0.001
SUA (μmol/L)	325.38 ± 93.59	317.99 ± 91.21	345.10 ± 97.52	−2.211	0.028

**Table 2:** Comparison between non PVH or mild PVH group and moderate severe PVH group.

Variables	Total number of cases (*n* = 290)	No or light (*n* = 186)	Moderate-severe (*n* = 104)	Test value	*p*-value
Age (years)	54.01 ± 12.52	50.30 ± 12.10	60.63 ± 10.37	−7.333	<0.001
Male [ex (%)]	201 (69.3)	124 (66.7)	77 (74.0)	1.704	0.192
History of hypertension [cases (%)]	196 (0.676)	110 (59.1)	86 (82.7)	16.890	<0.001
History of diabetes [cases (%)]	77 (26.6)	43 (23.1)	34 (32.7)	3.135	0.077
Smoking history [ex. (%)]	136 (46.9)	85 (45.7)	51 (49.0)	0.299	0.585
Drinking history [ex. (%)]	91 (31.4)	58 (31.2)	33 (31.7)	0.009	0.921
NIHSS rating	1.67 ± 1.03	1.64 ± 1.05	1.72 ± 0.99	−0.647	0.518
Infarct site [anterior circulation/case (%)]	193 (66.6)	121 (65.1)	72 (69.2)	0.523	0.470
TG (mmol/L)	1.61 ± 0.78	1.61 ± 0.82	1.62 ± 0.72	−1.155	0.877
HDL (mmol/L)	1.05 ± 0.28	1.05 ± 0.27	1.06 ± 0.29	−0.376	0.707
LDL (mmol/L)	2.65 ± 0.91	2.73 ± 0.94	2.51 ± 0.84	1.985	0.048
FPG (mmol/L)	5.31 (4.87 to 6.77)	5.26 (4.80–6.51)	5.45 (4.92–7.16)	−1.324	0.185
HbA1c (%)	6 (5.4–7.03)	6 (5.5–7)	6 (5.3–7.68)	−0.129	0.898
hs-CRP (mg/L)	1.91 (1.1 to 3.9)	1.8 (1 to 3.8)	2.15 (1.2 to 4.38)	−2.000	0.045
Hcy (μmol/L)	13 (9.7 to 17.63)	11.55 (9.1 to 16.1)	15.7 (11.5–23.15)	−4.870	<0.001
SUA (μmol/L)	325.38 ± 93.59	319.16 ± 89.92	336.49 ± 99.29	−1.516	0.131

### 3.2 The association between SUA and WMH

Two readers demonstrated a relatively high consistency in the WMH assessment, with *κ* = 0.783. Spearman correlation analysis showed that SUA level was positively correlated with DWMH (*r* = 0.123, *p* = 0.037), and there was no significant correlation between SUA level and PVH (*r* = 0.070, *p* = 0.234). As shown in Table 3, univariate logistic regression showed that old age (OR: 1.078, 95% CI: 1.051–1.106, *p* < 0.001), history of hypertension (OR: 2.835, 95% CI: 1.494–5.382, *p* = 0.001), high level of SUA (OR: 1.003, 95% CI: 1 to 1.006, *p* = 0.030) and homocysteine (OR: 1.033, 95% CI: 1.006 to 1.06, *p* = 0.016) were risk factors for moderate-to-severe DWMH. Advanced age (OR: 1.081, 95% CI: 1.055–1.107, *p* < 0.001), history of hypertension (OR: 3.301, 95% CI: 1.837–5.932, *p* < 0.001), high levels of homocysteine (OR: 1.05, 95% CI: 1.022 to 1.08, *p* = 0.001) were risk factors for moderate to severe PVH, but SUA level (OR: 1.002, 95% CI: 0.999 to 1.005, *p* = 0.132) did not significantly affect the severity of PVH. After adjusting for age, hypertensive history, and homocystine levels by multivariate logistic regression (Table 4), SUA level (OR: 1.005, 95% CI: 1.001–1.008, *p* = 0.008) was independently associated with DWMH severity, even after adjusting for the above confounders, SUA level (OR: 1.003, 95% CI: 1.000–1.006, *p* = 0.071) was not significantly associated with PVH.

**Table 3:** Univariate logistic regression analysis of risk factors influencing the severity of DWMH and PVH.

Factors	DWMH		PVH	
	OR (95% CI)	*p*-value	OR (95% CI)	*p*-value
Age	1.078 (1.051–1.106)	<0.001	1.081 (1.055–1.107)	<0.001
Gender	1.313 (0.738–2.338)	0.354	1.426 (0.836–2.432)	0.193
History of high blood pressure	2.835 (1.494 to 5.382)	0.001	3.301 (1.837–5.932)	<0.001
History of diabetes	0.916 (0.507 to 1.654)	0.771	1.615 (0.948–2.752)	0.078
Smoking history	1.146 (0.883 to 1.923)	0.606	1.143 (0.707–1.849)	0.585
Drinking history	0.864 (0.491 to 1.519)	0.611	1.026 (0.612–1.719)	0.923
NIHSS rating	1.206 (0.933 to 1.559)	0.152	1.081 (0.854–1.367)	0.517
Infarct site (anterior circulation)	1.034 (0.597 to 1.791)	0.906	1.209 (0.723–2.021)	0.470
TG	0.778 (0.538 to 1.127)	0.184	1.024 (0.755–1.39)	0.877
HDL	1.272 (0.507 to 3.192)	0.609	1.18 (0.5–2.785)	0.706
LDL	0.752 (0.552 to 1.024)	0.070	0.753 (0.568–1)	0.05
FPG	0.939 (0.837–1.052)	0.278	1.069 (0.974–1.174)	0.162
HbA1c	0.934 (0.806 to 1.082)	0.364	1.021 (0.899–1.16)	0.745
hs-CRP	1.005 (0.985 to 1.024)	0.635	0.999 (0.98–1.019)	0.939
Hcy	1.033 (1.006 to 1.06)	0.016	1.05 (1.022–1.08)	0.001
SUA	1.003 (1 to 1.006)	0.030	1.002 (0.999 to 1.005)	0.132

**Table 4:** Multivariate logistic regression analysis of risk factors affecting the severity of DWMH and PVH.

Factors	DWMH		PVH	
	OR (95% CI)	*p*-value	OR (95% CI)	*p*-value
Age	1.085 (1.054 to 1.117)	<0.001	1.085 (1.056–1.115)	<0.001
History of high blood pressure	1.790 (0.885 to 3.620)	0.105	2.327 (1.201–4.510)	0.012
Hcy	1.031 (1.001–1.061)	0.045	1.052 (1.022 to 1.084)	0.001
SUA	1.005 (1.002–1.008)	0.003	1.003 (1.000–1.007)	0.051

## 4 Discussion

This study investigated the relationship between SUA levels and the severity of WMH in patients with mild ischemic stroke, focusing on PVH and DWMH subtypes. The findings indicated a positive correlation between elevated SUA levels and the prevalence of DWMH, with higher SUA levels emerging as an independent risk factor for moderate to severe DWMH, even after adjustment for potential confounders. Conversely, no significant association was found between SUA levels and the severity of PVH when confounding factors were considered. This aligns with previous research (24, 25) on cerebral small vessel disease (CSVD), which suggests a linkage between uric acid levels and the progression of DWMH but not PVH, corroborating the results of the current study.

The mechanisms underlying the association between SUA and white matter hyperintensities (WMH) are multifaceted. One theory posits that high concentrations of SUA may cause the precipitation and deposition of uric acid microcrystals within vascular walls, thereby directly impairing the vascular endothelium and the blood–brain barrier (26). Additionally, uric acid is thought to provoke the production of various inflammatory mediators, leading to localized vascular inflammation and damage to the vascular intima (27). Another pathway might involve the interference of elevated SUA with lipid metabolism, promoting the oxidation of low-density lipoprotein (LDL) and accelerating cerebral atherosclerosis (28). Concurrently, high SUA levels may activate platelets, trigger the coagulation cascade, and promote thrombosis (29), resulting in occlusions of perforator arteries (30, 31) and contributing to demyelination in brain tissues, thereby elevating white matter signal intensity.

Our study demonstrates a selective association between SUA levels and DWMH, with no significant correlation observed for PVH. This differential association likely reflects distinct pathogenic mechanisms between these two WMH subtypes. Multiple histopathological investigations (11, 12) have suggested that cerebral small vessel atherosclerosis is the primary etiology of DWMH. Anatomically, the deep white matter receives blood supply from the medullary arteries originating from the cortical branches of the middle cerebral artery. This region is particularly susceptible to arteriosclerosis. Conversely, PVH is more commonly induced by chronic cerebral hypoperfusion and inadequate blood flow dynamics (13). Furthermore, in terms of venous drainage, DWMH solely relies on the deep medullary veins (DMV) within the venous system to drain arterial blood from the white matter. In contrast, PVH can be drained not only by the DMV but also by other subcortical veins. When the DMV sustains damage, the deep white matter, which depends solely on the DMV for drainage, is more severely affected and has a higher propensity to develop severe white matter lesions. PVH, on the other hand, can be compensated for by other veins and is thus relatively less impacted (32, 33). This explanation aligns with our results, indicating that elevated SUA levels promote lipid peroxidation as well as the oxidation of low-density lipoprotein and cholesterol. This, in turn, leads to increased blood lipid levels, accelerates the formation of atherosclerosis, and induces vascular damage, all of which contribute to the progression of DWMH. However, the impact of SUA on PVH is relatively more limited.

Additionally, demographic and clinical characteristics were analyzed, revealing that patients in the moderate to severe DWMH and PVH groups were older and had higher incidences of hypertension, elevated homocysteine levels, and hypersensitive C-reactive protein compared to those in the mild groups (*p* < 0.001 for all comparisons). The correlation between plasma homocysteine (Hcy) and WMH may be attributed to the pathophysiological effects of Hcy in the human body. Prior research has demonstrated that elevated Hcy levels can induce vascular wall alterations, with key mechanisms involving direct endothelial injury mediated by increased oxidative stress and pro-inflammatory responses (34, 35). Furthermore, basic experimental studies have confirmed that high concentrations of Hcy can activate N-methyl-D-aspartate (NMDA) receptors, thereby modulating cell adhesion and tight junctions, which subsequently leads to increased blood–brain barrier permeability (36). Elevated serum Hcy levels are associated with accelerated brain atrophy, ventricular dilation, and an increased risk of cerebrovascular diseases (37). A prospective study conducted among patients with atherosclerosis revealed that Hcy influences the progression of brain and kidney diseases, and is also linked to an increased risk of WMH lesions in the brain (38). Additionally, a retrospective analysis of 825 stroke patients indicated that Hcy is not only associated with large vessel atherosclerotic lesions but also serves as a critical determinant of small vessel diseases in the brain (39). Another study further corroborated that Hcy is significantly correlated with the severity of DWMH and PVH (40). These factors are recognized promoters of cerebral atherosclerosis and small-vessel disease, which includes WMH. These results further imply that SUA levels may be aligned with the alterations in these biomarkers, reinforcing the association between elevated SUA and the progression of WMH.

In conclusion, this study substantiates the role of SUA in the development of DWMH in patients with mild cerebral ischemia and underscores the potential of early SUA control and intervention in preventing cardiovascular and cerebrovascular diseases. Nonetheless, this study possesses several limitations, this study is a single-center, cross-sectional study with a small sample size. We hypothesized that the blood uric acid level is correlated with disorders of glucose and lipid metabolism, and that the history of diabetes and lipid levels may be important factors in the occurrence and development of WMH. However, in this study, we did not find any significance of their existence. Further large-sample studies are needed. We used the Fazekas scale for visual assessment of WMH, but human errors were inevitable. There were differences in the SUA detection times among different patients. The multivariate logistic regression analysis in this study may have residual confounding factors (such as lifestyle, genetic factors, etc.) that were not included in the analysis. The sample size of this study was determined based on the effect size estimation of similar studies and clinical feasibility. Future research will conduct in-depth power analysis.

## 5 Conclusion

In this study, higher serum uric acid levels were associated with an increased severity of deep white matter hyperintensities (DWMH) in patients with mild ischemic stroke, whereas no significant relationship was observed with periventricular hyperintensities (PVH). These findings suggest potential regional differences in the pathophysiology of white matter lesions. However, given the modest effect size and retrospective design, further prospective studies are needed to confirm these associations and evaluate their clinical relevance.

## Data Availability

The original contributions presented in the study are included in the article/supplementary material, further inquiries can be directed to the corresponding author.
